# Bta-miR-2400 Targets SUMO1 to Affect Yak Preadipocytes Proliferation and Differentiation

**DOI:** 10.3390/biology10100949

**Published:** 2021-09-22

**Authors:** Yongfeng Zhang, Lanhua Ma, Yarong Gu, Yongfang Chang, Chunnian Liang, Xian Guo, Pengjia Bao, Min Chu, Xuezhi Ding, Ping Yan

**Affiliations:** 1 Key Laboratory of Yak Breeding Engineering Gansu Province, Lanzhou Institute of Husbandry and Pharmaceutical Sciences, Chinese Academy of Agricultural Sciences, Lanzhou 730050, China; zhangyongfeng_ying@163.com (Y.Z.); MAlanhua666999@163.com (L.M.); Gu130511@163.com (Y.G.); muyaosuo@163.com (Y.C.); liangchunnian@caas.cn (C.L.); guoxian@caas.cn (X.G.); baopengjia@caas.cn (P.B.); chumin@caas.cn (M.C.); dingxuezhi@caas.cn (X.D.); 2 State Key Laboratory of Grassland Agro-Ecosystems, College of Pastoral Agriculture Science and Technology, Lanzhou University, Lanzhou 730000, China

**Keywords:** bta-miR-2400, yak adipocyte, SUMO1, proliferation, differentiation

## Abstract

**Simple Summary:**

Yak adipose tissue may have evolved a unique energy metabolism manner that accommodates the organism’s seasonal growth rhythms. Rare reports have shown that miRNAs regulate lipid metabolism in domestic yaks. In the present study, miR-2400 is a novel bovine miRNA; gain- and loss- function of bta-miR-2400 were performed in Yak preadipocytes, and it was revealed that bta-miR-2400 regulates lipid metabolism and energy homeostasis in yak preadipocytes by directly targeting small ubiquitin like modifier 1 (SUMO1) to promote cell proliferation and inhibit differentiation. These findings will undoubtedly facilitate future studies of miRNA regulation in lipid metabolism in high-altitude animals.

**Abstract:**

Yak adipose tissue may have evolved a unique energy metabolism manner to accommodate the organism’s seasonal growth rhythms. MiRNAs regulate multiple biological processes including systemic metabolism and energy homeostasis through post-transcriptional regulations. Rare reports have shown that miRNAs regulate lipid metabolism in domestic yaks. Therefore, we investigated the regulatory mechanisms of bta-miR-2400 in modulating yak preadipocytes proliferation and differentiation. We found that bta-miR-2400 was highly expressed in adipose tissue. Overexpression of bta-miR-2400 in yak preadipocytes significantly enhanced cell proliferation, increased the number of EdU fluorescence-stained cells, and promoted the expression of proliferation marker genes (*CDK2*, *CDK4* and *PCNA*). Besides, overexpression of bta-miR-2400 repressed the expression of adipogenesis-related marker genes, and the content of cellular triglyceride was substantially reduced. Conversely, inhibition of bta-miR-2400 showed opposite effects compared to those of bta-miR-2400 overexpression in yak preadipocytes. Further, luciferase reporter assays revealed that SUMO1 is a target gene of bta-miR-2400, with bta-miR-2400 being able to down-regulate SUMO1 mRNA and protein expression. In conclusion, bta-miR-2400 regulates lipid metabolism and energy homeostasis in yak preadipocytes by directly targeting SUMO1 to promote cell proliferation and inhibit differentiation.

## 1. Introduction

The yak (*Bos grunniens*) is an animal of economic importance living in the adjacent areas of the Qinghai-Tibetan Plateau and is the only livestock resource that can make full use of natural pasture resources for animal production, and its wool, meat, milk and other by-products provide an effective livelihood and economic source for local nomadic herders [1,2,3]. Yak may have evolved a special way of metabolizing energy to survive in the extreme conditions of long cold seasons and malnutrition on the Qinghai-Tibetan Plateau [4,5]. Aside from skeletal muscle, adipose tissue is another important factor affecting the meat quality of livestock [6]. In livestock and poultry, the fat excessive deposition will affect the carcass quality and the health of consumers. Adipocytes are the main component of adipose tissue and their proliferation and differentiation are regulated by a complex and orderly molecular network, with a large number of transcription factors and miRNAs [7,8].

As endogenous non-coding RNAs, miRNAs are found in the genomes of plants and animals in the form of single-copy, multi-copy and gene clusters [9,10]. They are involved in the growth and development of different species, tissues, organs, and participated in the regulation of gene expression through specific binding to degrade target mRNA or block the post-transcriptional translation of target mRNAs [11]. Many studies have confirmed that miRNAs are involved in the molecular regulation of adipose tissue development and lipid metabolism. For example, Karbiener et al. found that overexpression of miR-27b reduced the ability to induce differentiation for PPARγ and C/EBPα, and miR-27b specifically bound *PPARγ* 3′-UTR to inhibit 3T3-L1 adipocyte differentiation [12]. Bta-miR-149-5p represses bovine adipocyte proliferation and differentiation by targeting *CRTCs* both at the transcriptional and post-transcriptional levels [13]. Ma et al. revealed that miR-130 a/b reduced lipid droplet accumulation and inhibited bovine preadipocyte differentiation through targeting *PPARG* and *CYP2U1* [14]. Besides, miRNAs were found to affect lipid metabolism by participating in many biological pathways. Mi et al. showed that miR-139-5p binds to the 3′UTR region of *IRS1* and inhibits differentiation and promotes proliferation of 3T3-L1 adipocytes via the PI3K/Akt signaling [15]. A miRNA expression profiling microarray study disclosed that miR-210, miR-148a and other 18 miRNAs promoted adipocyte differentiation by inhibiting the Wnt/β-catenin signaling pathway [16]. MiR-210 was further reported to target *TCF7L2* to inhibit Wnt/β-catenin signaling, thereby promoting adipogenesis. Likewise, miR-181a-5p targets and downregulates *TCF7L2* expression to increase C/EBPα and PPARγ expression, promoting adipogenesis while inactivating Wnt-regulated gene transcription by binding to β-catenin [17]. These studies demonstrate that miRNAs play an essential role in adipogenesis and fat metabolism. 

Interestingly, bta-miR-2400 is a bovine-specific miRNA whose sequence cannot be mapped to other mammalian genomes [18]. It is located at 33164739~333164812 of BAT27 and inside the eighth intron of WHSC1L1 gene [19]. Besides, we found that bta-miR-2400 was highly expressed in yak adipose tissue compared to other tissues. Therefore, we hypothesized that bta-miR-2400 may play a role in yak adipose tissue development. Further, gain- and loss-of-function studies were used to elucidate the molecular mechanism by which bta-miR-2400 governs yak preadipocytes proliferation and differentiation. The present study may provide a theoretical foundation for further investigations into miRNA regulatory mechanisms in yak preadipocyte adipogenesis.

## 2. Materials and Methods

### 2.1. Animals and Sample Collection

Animal handling in the study was approved by the Committee of Animal Management and Ethics in the Lanzhou Institute of Husbandry and Pharmaceutical Sciences, Chinese Academy of Agricultural Sciences, China. (permit number: SYXK-2014-0002). Experimental animals were provided by Datong Yak Breeding Center (Datong, Qinghai, China). Tissues (Heart, liver, spleen, lung, kidney, muscle and adipose tissue) were obtained from three affinity-free female yaks at 18 (98, 92 and 97 kg) and 36 (154, 156 and 159 kg) months. Samples were rapidly frozen in liquid nitrogen.

### 2.2. The Isolation and Induced Differentiation of Yak Preadipocytes

Primary yak preadipocytes were isolated from yak perirenal adipose tissue according to previous studies [20,21]. Briefly, the adipose tissue was cut into approximately 1 mm^3^ pieces under aseptic conditions and digested with type I collagenase at 37 °C for 60~90 min with continuous shaking. The digestion reaction was then terminated by adding growth medium containing 15% fetal bovine serum (FBS) (HyClone, Thermo Fisher Scientific, Carlsbad, CA, USA), and the digestion mixture was filtered through 70 μm and 40 um strainers, respectively. The filtrate was transferred to 15 mL centrifuge tubes, centrifuged at 1500× *g* for 10 min, and the precipitate was washed twice with serum-free DMEM-F12 (HyClone, Thermo Fisher Scientific, Carlsbad, CA, USA). Subsequently, the cells pellet was resuspended in DMEM-F12 growth medium containing 15% fetal bovine serum and seeded into 25 cm^2^ cell culture flasks incubated at 37 °C, 5% CO_2_.

### 2.3. Cell Culture and Transfection

Yak preadipocytes were seeded in 12-well plates at a density of 1 × 10^4^ cells/well and cultured with DMEM-F12 growth medium (10% FBS, 1% penicillin-streptomycin) at 37 °C, saturated humidity and 5% CO_2_. Cells were cultured with Opti-MEM medium (1.5 mL per well) for 4 h without antibiotics before differentiation when the cell density reached about 80%, followed by infecting the preadipocytes with bta-miR-2400 mimic, negative control (Ribobio, Guangzhou, China) at a concentration of 50 nM, bta-miR-2400 inhibitor (Ribobio, Guangzhou, China) at a concentration of 100 nM using lipofectamine 3000 (Invitrogen, Carlsbad, CA, USA).

### 2.4. Cell Induced Differentiation and Oil Red O Staining

The cells were seeded into 6-well plates as 1 × 10^4^ cells/well, and cultured in DMEM/F12 growth medium (shown above) at 37 °C, saturated humidity and 5% CO_2_. The medium was changed every two days. When it comes to cell fusion, the growth medium was changed to an induction differentiation medium containing 10% FBS, 0.5 mmol/L 3-isobutyl-methylxanthine (Sigma, Santa clara, CA, USA), 1 μmol/L dexamethasone (Sigma, Santa clara, CA, USA), and 10 μg/mL insulin (Sigma, Santa clara, CA, USA). The induction differentiation medium was then changed to a maintenance medium (10% FBS, 10 μg/mL insulin) after two days until cells were completely differentiated. The cells were washed twice with pre-warmed PBS, and fixed with 4% paraformaldehyde for 1 h at room temperature. After fixation, cells were washed with PBS three times, and stained with Oil Red O working solution for 30 min at room temperature, followed by the removal of the excess staining solution and microscopy.

### 2.5. Triglyceride Assay

Yak preadipocytes were inoculated into 12-well plates at a density of 1 × 10^4^ cells/well. Bta-miR-2400 mimics, inhibitors, and negative control (NC) (Ribobio, Guangzhou, China) transfected cells were collected at day 12 of induction differentiation. The cells were washed twice with PBS and scraped into 1.5 mL EP tubes. The cells were spun down at 1000 g for 10 min, after which the supernatant was carefully removed and the cell pellet was resuspended with 200 μL PBS. The cell suspension was then ultrasonically crushed for 1 min in ice to obtain samples for the triglyceride assay. Triglyceride content was measured according to the protocol of the commercial kit (Meilian, Shanghai, China).

### 2.6. CCK-8 Proliferation Assay

Yak preadipocytes were seeded in 96-well plates at a density of 1 × 10^3^ cells/well and transfected with bta-miR-2400 mimic, NC, bta-miR-2400 inhibitor until cell confluent reached 70–80%. Cell proliferation was measured at 12 h, 24 h and 48 h after cell transfection by CCK-8 (Beyotime, Shanghai, China). Cells were incubated with 10 μL CCK-8 reagents for 1 h at 37 °C, 5% CO_2_, and the absorbance values were measured by Multiskan FC at 450 nm (Thermo, Waltham, MA, USA).

### 2.7. EdU Proliferation Assay

Yak preadipocytes were seeded into 24-well plates and transfected with bta-miR-2400 mimic, NC, bta-miR-2400 inhibitor when the cell density reached approximately 50%. Cell proliferation was measured using the EdU-488 cell proliferation assay kit (Beyotime, Shanghai, China) according to the manufacturer’s procedures.

### 2.8. qRT-PCR Analysis

Cellular and tissue RNA was extracted by TRIzol (Invitrogen, Carlsbad, CA, USA). The mRNA cDNA and miRNA cDNA were synthesized by mRNA and miRNA reverse transcription kits (all purchased from Takara, Dalian, China), respectively. For qPCR assay, three biological and two technical replicates were performed in the present study. Relative expression of bta-miR-2400 and genes was detected by SYBR Premix Ex Taq kit (TaKaRa, Dalian, China) using a CFX96^TM^ Real-Time system (Bio-Rad, Hercules, CA, USA), U6 and *β-Actin* were used for miRNA and mRNA normalization, respectively. The qPCR reaction condition is shown below: denaturation 30 s at 95 °C, followed by 40 cycles at 94 °C for 15 s and annealing at 72 °C for 30 s, the melt curve was generated at 95 °C with a heating rate of 0.5 °C per 10 s. The expression of genes and miRNA were performed using 2^−ΔΔCt^. The primer sequences are shown in Table 1.

### 2.9. Luciferase Reporter Assay

TargetScan (http://www.targetscan.org/vert_72/ (accessed on 23 August 2021)) and miRBase (https://www.mirbase.org/ (accessed on 23 August 2021)) were used to predict the target genes of bta-miR-2400. SUMO1 was a potential target gene of bta-miR-2400 based on the prediction. Wild type 3′UTR of SUMO1 (about 200 bp) harboring the bta-miR-2400 binding site, as well as the binding site mutated sequence, were cloned into pmirGLO vector (Promega, Madison, WI, USA), referred to as SUMO1-WT and SUMO1-MT, respectively. The reporter vectors were transfected into 293T cells together with bta-miR-2400 mimics or NC using Lipofectamine 3000. The dual-luciferase assay was applied using Luciferase Reporter Assay Kit (Promega, Mannheim, Germany) at 48 h after transfection. The primer sequences used for cloning are presented in supplementary Table S1.

### 2.10. Western Blot Analysis

Cells were collected in 1.5 mL EP tubes and the cellular total protein was extracted using a Total Protein Extraction Kit (Beyotime, Shanghai, China) according to the kit instructions. Protein concentration was measured using a BCA protein assay kit (Beyotime, Shanghai, China). The protein of equal amounts (~15 µg) was denatured at 95 °C for 15 min with a sample loading buffer. Samples were then separated by sodium dodecyl sulfate-polyacrylamide electrophoresis using 10% separator gel at 100 V for 80 min and 4% concentrator gel at 40 V for 25 min. Protein bands were transferred from the gel to the PVDF membrane (Bio-Rad) and the membranes were then incubated in monoclonal rabbit anti-SUMO1, rabbit anti-PPARγ and mouse anti-β-Actin (1:1000; Abcam, Cambridge, UK) overnight at 4 °C. Further, the membrane was washed three times with TBST on a decolorizing cradle for 5 min per time, and incubated with goat anti-rabbit IgG (1:5000; Beyotime, Shanghai, China) labeled with HRP for 30 min at room temperature. Finally, the membranes are rinsed three times with TBST to detect the chemiluminescence signal by exposing membranes and photographing with the Bio-Rad Chemi Doc system. Grayscale values of proteins were evaluated by Image J (https://imagej.nih.gov/ij/ (accessed on 23 August 2021)).

### 2.11. Statistical Analysis

Statistical analysis was performed using Statistical Product and Service Solutions (SPSS) 24.0 software. The difference between experimental and control groups was tested by one-way analysis of variance (ANOVA), and Duncan’s Multiple Range Test was used for multiple comparisons, * *p* < 0.05, ** *p* < 0.01 indicated statistical significance, results were indicated as means ± SEM.

## 3. Results

### 3.1. Expression of Bta-miR-2400 in the Yak Tissues and Adipocytes

**Figure 1:**
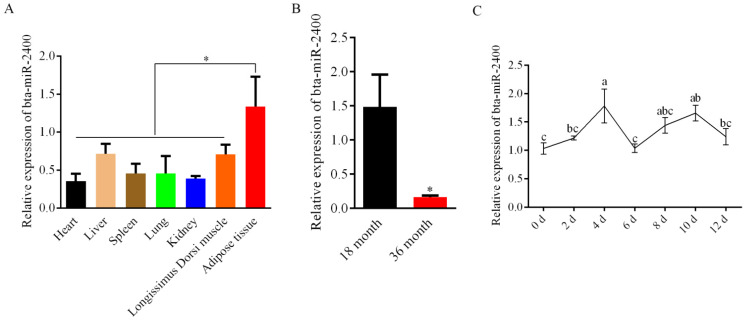
Expression profiles of bta-miR-2400 in tissues and adipocytes. (**A**) The bta-miR-2400 expression in different yak tissues and (**B**) different adipose tissue stages. (**C**) The expression pattern of bta-miR-2400 during yak preadipocyte differentiation. * *p* < 0.05. Duncan’s Multiple Range Test was used for multiple comparisons, different lowercase letters represent significant differences (*p* < 0.05).

The quantitative real-time PCR was employed to detect the expression of bta-miR-2400 in a variety of tissues. By comparing the expression of bta-miR-2400 in different yak tissues, we found that bta-miR-2400 was highly expressed in adipose tissue (Figure 1A), and bta-miR-2400 expression was sharply decreased at 36 months compared to 18 months in adipose tissue (Figure 1B). Further, we examined the expression of bta-miR-2400 during the yak preadipocytes differentiation. The expression of bta-miR-2400 increased and peaked on day 4, then decreased during the yak preadipocytes differentiation (Figure 1C). The above findings indicate that bta-miR-2400 may play a role in yak preadipocyte proliferation and differentiation.

### 3.2. Bta-miR-2400 Promotes the Proliferation of Yak Preadipocytes

**Figure 2:**
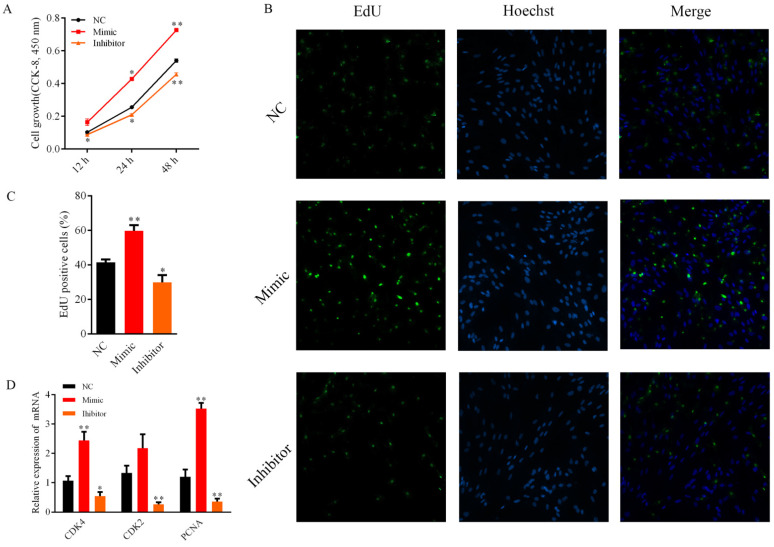
Bta-miR-2400 promotes yak preadipocyte proliferation. Cells were transfected with bta-miR-2400 mimic, NC, and inhibitor. (**A**) Cell Counting kit 8 (CCK-8) assays and (**B**,**C**) 5-ethynyl-20-deoxyuridine (EdU) proliferation were performed to measure cell proliferation (*n* = 6 per treatment per time point, magnification 200×); (**D**) Relative expression of cyclin-dependent kinases 2 (CDK2), cyclin-dependent kinases 4 (CDK4) and proliferating cell nuclear antigen (PCNA). * *p* < 0.05; ** *p* < 0.01.

To investigate the function of bta-miR-2400 on the proliferation of yak preadipocytes, cells were transfected with bta-miR-2400 mimic, NC, and inhibitor, respectively. The CCK-8 assay showed that the proliferation viability of the bta-miR-2400 mimic-transfected cells was enhanced compared to the NC (Figure 2A). On the contrary, cell proliferation viability was dramatically reduced in the bta-miR-2400 inhibitor group than in the NC (Figure 2A). Furthermore, the effect of bta-miR-2400 on the proliferation of yak preadipocytes was further confirmed by EdU assay. Overexpression (mimic) of bta-miR-2400 significantly increased the number of EdU-labeled cells (Figure 2B,C); inhibition (inhibitor) of bta-miR-2400 significantly decreased the number of EdU-labeled cells (Figure 2B,C). Cell cycle-related genes (CDK4 and PCNA) were significantly (*p* < 0.01) increased after transfection with bta-miR-2400 mimic compared to the control (NC) (Figure 2D), and the expression of CDK2, CDK4 and PCNA were significantly (*p* < 0.05) down-regulated after transfection with bta-miR-2400 inhibitors (*p* < 0.05) (Figure 2D). These findings suggest that bta-miR-2400 promotes the proliferation of preadipocytes.

### 3.3. Role of Bta-miR-2400 during Yak Preadipocytes Lipogenic Differentiation

**Figure 3:**
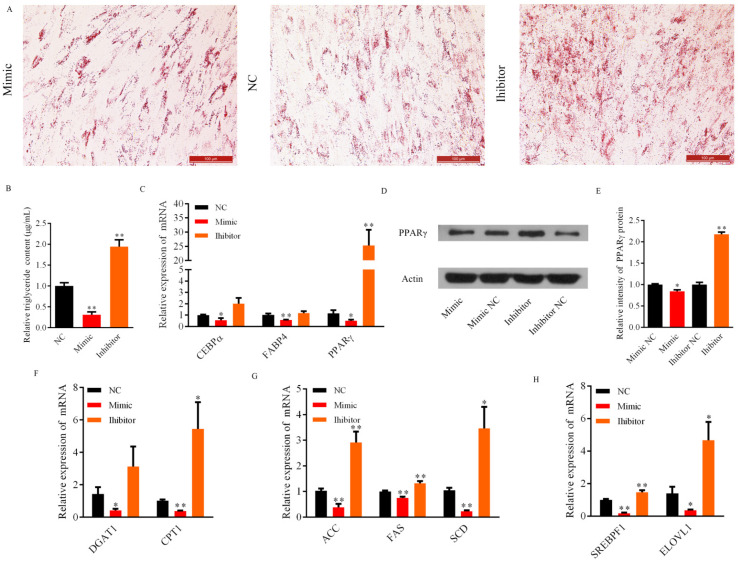
Bta-miR-2400 inhibits yak preadipocyte differentiation. Yak preadipocytes were transfected with bta-miR-2400 mimics, inhibitors or negative control (NC) for 10 days after induced differentiation. (**A**) Adipocytes were stained with Oil red O. (**B**) The cellular triglyceride content was analyzed by spectrophotometer. (**C**) Expression levels of adipogenic markers (*PPARγ*, *FABP4*, and *C/EBPα*). (**D**,**E**) The protein level of PPARγ in adipocytes transfected with bta-miR-2400 mimics, inhibitors and negative control. The expression of genes related to (**F**) fatty acid transportation (*DGAT1*, *CPT1*), (**G**) fatty acid synthesis (*ACC*, *SCD* and *FAS*), and (**H**) lipogenic transcription (*ELVOL*, *SREBPF1*). * *p* < 0.05; ** *p* < 0.01.

The proliferation and differentiation are highly orchestrated events in cell development [22]. Therefore, this research explored the role of bta-miR-2400 in yak preadipocytes adipogenesis. Interestingly, the Oil Red O staining assay revealed that overexpression of bta-miR-2400 significantly inhibits yak preadipocyte differentiation (Figure 3A). Moreover, the triglyceride content was reduced in the adipocytes transfected with bta-miR-2400 mimic (Figure 3B). Accordingly, triglyceride content was significantly higher in the group transfected with bta-miR-2400 inhibitors compared to the control group (Figure 3B). Meanwhile, we detected the expression of adipogenesis-related genes and found that the expression of adipogenic marker genes (PPARγ, C/EBPα and FABP4) (Figure 3C), fatty acid transport-related genes (CPT1 and DGAT1) (Figure 3F), fatty acid synthesis-related genes (ACC, FAS and SCD) (Figure 3G), and adipogenic transcription-related genes (ELVOL1 and SREBPF1) (Figure 3H) were significantly decreased (*p* < 0.05) in the bta-miR-2400 mimics experimental group compared to the NC. The expression of PPARγ, FABP4, ELVOL1, SREBPF1, CPT1, DGAT1, FAS, ACC and SCD were elevated in the group transfected with bta-miR-2400 inhibitors compared to NC (Figure 3C,F–H). Interestingly, the PPARγ expression was dramatically higher in the inhibitor group compared to NC (Figure 3C). As a key transcriptional regulator of adipogenesis in animals, PPARγ directly modulates the expression of adipocyte differentiation related genes and lipid metabolism [23]. Thus, we also examined the impact of bta-miR-2400 on PPARγ protein expression. Overexpression or downregulation of bta-miR-2400, PPARγ protein levels were reduced or increased compared to NC, respectively (Figure 3D,E; Figure S1). Collectively, these findings demonstrated that bta-miR-2400 inhibited the differentiation of yak preadipocytes.

### 3.4. Bta-miR-2400 Directly Targets the 3′UTR of SUMO1

**Figure 4:**
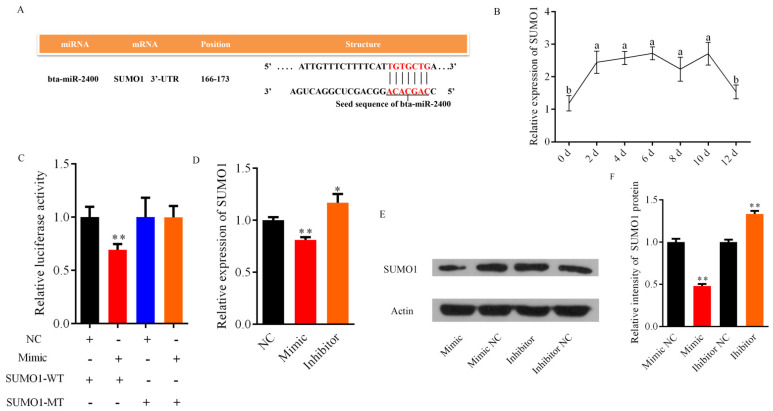
Bta-miR-2400 targets the 3′UTR of SUMO1. (**A**) The schematic of the sequence alignment between bta-miR-2400 and 3′UTR of SUMO1. (**B**) The expression pattern of bta-miR-2400 during yak adipocyte differentiation. (**C**) Luciferase reporter assay for bta-miR-2400 and 3′UTR of SUMO1. (**D**–**F**) Expression of SUMO1 mRNA and protein for cells transfected with bta-miR-2400 mimics, inhibitors and negative control. * *p* < 0.05; ** *p* < 0.01. Different lowercase letters represent significant differences (*p* < 0.05).

Bioinformatic analysis was performed using miRBase and TargetScan online tools, and the prediction revealed that the small ubiquitin like modifier 1 (SUMO1) is one of the potential target genes of bta-miR-2400, SUMO1 (Accession number: XM_005904253.2) contains a specific binding site for bta-miR-2400 at 166–173 bp within its 3′UTR (Figure 4A). The expression of SUMO1 peaked at day 6 of yak preadipocytes, and then decreased at day 12 (Figure 4B). Thus, we hypothesized that SUMO1 was targeted by bta-miR-2400, luciferase assay was then used to address this point. The luciferase activity was significantly decreased in the group co-transfected with SUMO1-3′UTR-WT and bta-miR-2400 mimic compared to the control group (SUMO1-3′UTR-WT + mimic NC; SUMO1-3′UTR-MT + bta-miR-2400 mimic). There was no significant difference of luciferase activity between the SUMO1-3′UTR-MT + mimic NC and SUMO1-3′UTR-MT + mimic groups (Figure 4C). Further, both SUMO1 mRNA and protein were significantly decreased (*p* < 0.01) in the bta-miR-2400 mimics group compared to the NC group (Figure 4D–F; Figure S1). On the country, the expression of SUMO1 mRNA and protein were markedly (*p* < 0.05) increased in the bta-miR-2400 inhibitor group compared to the NC group (Figure 4D–F; Figure S1). These data confirmed that SUMO1 is the direct target gene of bta-miR-2400.

## 4. Discussion

Yak is a dominant herbivorous species distributed on the Qinghai-Tibet Plateau and its adjacent areas. Yak body fat fluctuates greatly during the annual growth cycle due to environmental factors such as terrain, pasture biomass and climate [24]. As a result of pasture being abundant in the warm season (June to September), yaks rapidly accumulate adipose tissue to maintain body temperature and adequate energy metabolism during the long cold season (October to May) [25]. The adipose tissue development cannot be separated from two important biological processes of preadipocytes proliferation and differentiation, and miRNAs are reportedly involved in this important process [26,27]. Nevertheless, a clear understanding of miRNAs in the regulation of cellular metabolism in yaks remains elusive. Interestingly, bta-miRNA-2400 is a bovine specific miRNA. Furthermore, the expression level of bta-miRNA-2400 was significantly higher in adipose tissue compared to other tissues. Also, to explore the regulatory mechanism of miRNAs on yak adipose energy metabolism balance, miRNA gain- and loss-function experiments were applied in this study. On one hand, bta-miRNA-2400 can promote the proliferation of yak preadipocytes that was evidenced by cell counting, EdU and mRNA expression analysis with proliferation-related marker genes. On the other hand, the effect of bta-miRNA-2400 for preadipocyte differentiation was confirmed by Oil red O staining, triglyceride content, and the expression levels of adipogenesis-related genes and protein.

Cyclin-dependent kinases regulate the proliferation of mammalian cells, each containing a catalytic subunit (CDK) and a regulatory subunit (cyclin) [22]. CDK2 and CDK4 are important components of cell cycle-dependent kinases (CDKs) that govern the entry for the G1-to-S phase of the cell cycle [28,29]. PCNA is the link between CDK2 and its action substrate and phosphorylated by CDK2 kinase, thereby promoting cell proliferation [30]. Therefore, the expression of *CDK2*, *CDK4* and *PCNA* was detected after the cell transfected with bta-miRNA-2400 mimics, inhibitors, or NC, respectively. Overexpression of bta-miR-2400, CCK-8 and EdU data showed that the cell proliferation rate was accelerated, while the findings revealed that the expression of *CDK4* and *PCNA* were significantly increased. Conversely, inhibition of bta-miR-2400 decreased the expression of these genes in addition to inhibiting cell proliferation. These results indicated that bta-miR-2400 promotes the proliferation of yak preadipocytes.

Another important function of miRNA is to regulate cell differentiation [27]. Therefore, we explored the role of bta-miR-2400 during yak preadipocytes differentiation. In the current study, we examined the expression of fatty acid regulatory genes when cells were transfected with bta-miR-2400 mimics, inhibitors, or NC. Compared to the NC group, marker genes for adipogenic differentiation (*PPARγ*, *C/EBPα* and *FABP4*), fatty acid transport-related genes (*CPT1* and *DGAT1*), fatty acid synthesis-related genes (*ACC*, *FAS* and *SCD*), and adipogenic transcription-related genes (*ELVOL1* and *SREBPF1*) were down-regulated in the bta-miR-2400 mimic group. In contrast, the inhibition of bta-miR-2400 had opposite effects on the expression of fatty acid regulatory genes compared to overexpression of bta-miR-2400. Specifically, the adipogenic marker gene PPARγ was expressed at consistent levels of protein and mRNA in different groups. As the most important transcription factor for lipogenic differentiation, PPARγ activates downstream target genes related to adipocyte proliferation and differentiation [31,32]. It implies that bta-miR-2400 may impair the differentiation of yak preadipocytes by affecting the PPARγ expression. Additionally, the analysis of Oil red O staining and triglyceride content revealed that the lipid droplets and triglyceride content were decreased in yak preadipocytes transfected with bta-miR-2400 mimics. These findings indicated that bta-miR-2400 may play as a negative regulator for yak preadipocyte differentiation.

Previous studies have identified that miRNAs directly act on the 3′UTR of their target genes to regulate adipocyte proliferation and differentiation [12,33,34]. For instance, bta-miR-1271 promotes differentiation of bovine preadipocytes via targeting the activating transcription factor 3 (*ATF3*) [35], and bta-miR-224 suppresses lipogenic differentiation of bovine preadipocytes by targeting *LPL* [36]. SUMO1 was predicted as a target gene of bta-miR-2400, and previous studies revealed that SUMO1 was associated with adipogenesis [37]. Therefore, SUMO1 was assumed to be involved in the proliferation and differentiation of yak preadipocytes. In the present study, dual-luciferase reporter assays verified a direct targeting relationship between bta-miR-2400 and SUMO1. Besides, qRT-PCR and western blot analysis indicated that SUMO1 expression was repressed by overexpression of bta-miR-2400, and SUMO1 expression was rescued by inhibition of bta-miR-2400. The current results are in line with previous studies that found that miRNAs typically restrain gene expression in animals by binding to the 3′UTR of the target gene [38].

SUMO1 (small ubiquitin like modifier 1) is a ubiquitin-related modifier protein that plays a post-translational modification role by attaching to other proteins in a general and reversible manner during a variety of cellular processes and the development of organisms [37]. It found that the aggregation (sumoylation) of SUMO1 affects subcellular localization and stability of substrate, therefore affecting their transcriptional activity [39]. Besides, Laura et al. found that adipogenesis was abnormal for SUMO1-deficient mice [37]. In addition, both SUMO1-deficient cells and mice showed an attenuated response to the PPARγ agonist rosiglitazone. These findings suggest that SUMO1 regulates adipogenesis and has an important role in the transcriptional activation of the PPAR γ signaling pathway. PPARγ is a decisive transcription factor for adipocyte differentiation, and its function is modulated by various post-translational modifications, including phosphorylation, SUMOylation, ubiquitination, acetylation, and O-GlcNAcylation [40]. For SUMOylation, SUMO1 affects the stability and transcriptional activity of PPARγ [41]. It was found that PPARγ possesses two functional SUMO sites, lysine 77 in the activation function (AF) 1 domain and lysine 365 in the activation function (AF) 2 domain of PPARγ1, and lysine 107 in the AF1 domain and lysine 395 in the AF2 domain of PPARγ2, which regulate the activity of PPARγ by binding to these sites [42,43,44]. Specifically, adipocytes are unable to continue differentiation in the absence of PPARγ [39]. Surprisingly, the mRNA and protein expression pattern of PPARγ and SUMO1 in the present study were essentially the same in different groups. Consequently, it is inferred that bta-miR-2400 may attenuate the binding of SUMO1 to the functional sites of PPARγ by negatively regulating SUMO1 expression, therefore diminishing the activation of SUMO1 for PPARγ stability and transcriptional activity. 

## 5. Conclusions

In summary, bta-miR-2400 both promotes the proliferation of and inhibits the differentiation of yak preadipocytes by directly targeting the 3′UTR of SUMO1. The present study has provided us with a deeper understanding of the miRNAs role in the proliferation and differentiation of yak adipocytes and laid the foundation for comprehension of its regulatory mechanisms in bovine lipid metabolism.

## Figures and Tables

**Table 1:** Sequence specific primers were used in this study. F: forward, R: reverse. β-Actin and U6 were used as endogenous control genes for mRNA and miRNA.

Gene	Primer Sequence (5′ to 3′)	Tm/°C	Product Size/bp	Accession Numbers
*SCD*	F:CTGGTGTCCTGTTGTTGTGC R:GGTCTTGTCATAAGGGCGGT	60.0	179	XM_005892055.2
*PPAR γ*	F:CATTTCCACTCCGCACTA R:GGGATACAGGCTCCACTT	60.3	122	XM_005902846.1
*C/EBPα*	F:GGCATCTGCGAACACGAGA R:AGGAACTCGTCGTTGAAGGC	60.3	212	XM_005893918.2
*FABP4*	F:CATTAAATCCGAAAGCA R:CTCATAAACTCTGGTGGC	60.3	242	XM_014478668.1
*FAS*	F:GCAAAGTGGTCATTCAGGTACG R:CCCAGTGATGATGTAGCTCTTG	64.5	125	NM_001012669.1
*ACC*	F:GAGACAAACAGGGACCATT R:AGGGACTGCCGAAACAT	61.4	142	AJ132890.1
*CPT1*	F:TCGACCCAAACAAGTACCCC R:CGCTGGGCATTTGTCTCTGA	60.0	157	NM_001034349.2
*DGAT1*	F:GAGACAAACAGGGACCATT R: AGGGACTGCCGAAACAT	64.5	92	JF913457.1
*SUMO1*	F:CATTGGACAGGATAGCA R:CTCCATTCCCAGTTCTT	59.0	172	NM_001035458.1
*ELOVL1*	F:AGAAAGACGGACAGGTGAC R:GCAGGAAGTAGTATTGGGAG	61.4	273	XM_010803685.3
bta-miR-2400	F:CCAGCACAGGCAGCTCGGACTGA	60.3	_	
*SREBPF1*	F:GCTGACCGACATAGAAGACA R:CTCATCGTGGAAGGAGGTGG	61.0	165	NM_001113302.1
*PCNA*	F:TCCGAGGGCTTCGACACTTA R:GTGCCAACGTGTCTGCGTTA	63.3	142	XM_005906528.2
*CDK2*	F:AGGTGGTGACTCTGTGGTA R:GCTCCGTCCATCTTCAT	59.0	299	XM_005905862.2
*CDK4*	F:TTGGTGTCGGTGCCTAT R:TCCAGACGCCGCAGTAA	57.0	157	XM_005901664.1
*U6*	F:GGAACGATACAGAGAAGATTAGC R:TGGAACGCTTCACGAATTTGCG	60.3	_	
*β-Actin*	F:GCAGGTCATCACCATCGG R:CCGTGTTGGCGTAGAGGT	60.3	158	XM_005887322.2

## Data Availability

The data presented in this study are available in the present study and Supplementary Material.

## Supplementary Materials

The following are available online at https://www.mdpi.com/article/10.3390/biology10100949/s1, Table S1: Primers for the dual-luciferase reporter assay. F: forward, R: reverse. Figure S1: The western blot original image included PPARγ and SUMO1.
