# Supplementary material for: Bta-miR-2400 Targets SUMO1 to Affect Yak Preadipocytes Proliferation and Differentiation
Source: Biology (Basel). 2021 Sep 22;10(10):949. doi: 10.3390/biology10100949 (PMC8533534; doi:10.3390/biology10100949)
Supplement: Supplementary file 1 [file biology-10-00949-s001.zip › biology-1365675 supplementary/biology-1365675 Figure S1.pdf]

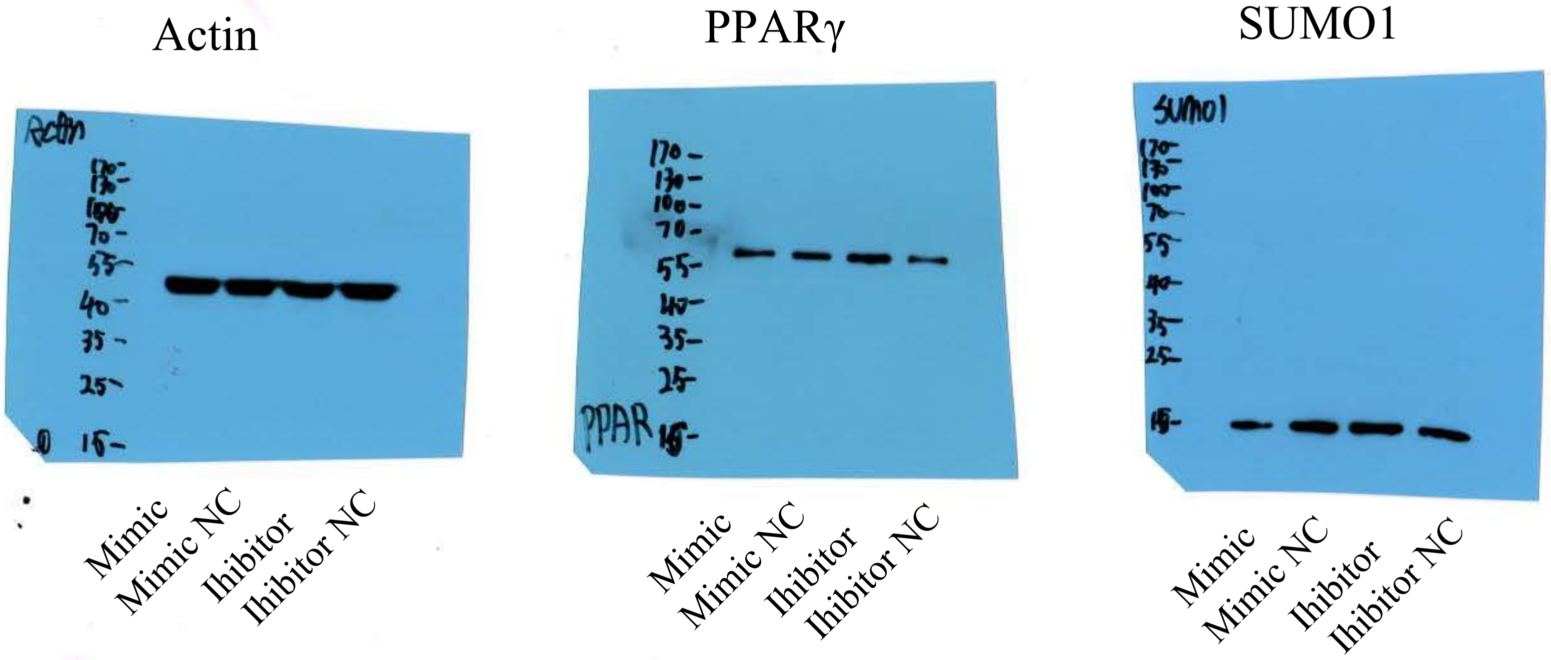
Actin
PPARγ
SUMO1
Mimic
Mimic NC
Ihibitor
Ihibitor NC
Mimic
Mimic NC
Ihibitor
Ihibitor NC
Mimic
Mimic NC
Ihibitor
Ihibitor NC
