# Supplementary material for: Bta-miR-2400 Targets SUMO1 to Affect Yak Preadipocytes Proliferation and Differentiation
Source: Biology (Basel). 2021 Sep 22;10(10):949. doi: 10.3390/biology10100949 (PMC8533534; doi:10.3390/biology10100949)
Supplement: Supplementary file 1 [file biology-10-00949-s001.zip › biology-1365675 supplementary/biology-1365675-Table S1.pdf]

Table S1. Primers for the dual-luciferase reporter assay. F: forward, R: reverse.

Name	Primer sequence (5' to 3')
SUMO1-WT	F1:CTAGTTGTTTAAACGAGCTCGCTAGCTGTAATGTGGTGTTCAAAACATAATTGAAACTG<br>R1:GAATTCAAATTACCAGATGTTTTAAAGAGATGGGGTGCCAGTTTCAATTATGTTT
SUMO1-WT	F2:TCTGGTAATTTGAATTCTAGTGCCCATTATTCATTATTGTTTCTTTTCATTGTGC<br>R2:TGATATGAAGGGGGCTGAGGTTTGATCACCAAAAATCAGCACAATGAAAAGAAAC
SUMO1-WT	F3:CAGCCCCCTTCATATCACTCTCTCCTTTTTAAAAATTACATGTGTGCATAGAGAG<br>R3:GCCTGCAGGTCGACTCTAGACTCGAGCCTGGAAAAGGCGGGCTCTCTATGCACACATG
SUMO1-MT	F1:CTAGTTGTTTAAACGAGCTCGCTAGCTGTAATGTGGTGTTCAAAACATAATTGAAACTG<br>R1:GAATTCAAATTACCAGATGTTTTAAAGAGATGGGGTGCCAGTTTCAATTATGTTT
SUMO1-MT	F2:TCTGGTAATTTGAATTCTAGTGCCCATTATTCATTTAACAAAGAAAAGTAACACG<br>R2:TGATATGAAGGGGGCTGAGGTTTGATCACCAAAAACGTCGTGTTACTTTTCTTTG
SUMO1-MT	F3:CAGCCCCCTTCATATCACTCTCTCCTTTTTAAAAATTACATGTGTGCATAGAGAG<br>R3:GCCTGCAGGTCGACTCTAGACTCGAGCCTGGAAAAGGCGGGCTCTCTATGCACACATG
